# Prognostic value of functional capacity assessed by the Glittre Activities of Daily Living test in hemodialysis patients: a prospective observational study

**DOI:** 10.1590/2175-8239-JBN-2025-0316en

**Published:** 2026-09-11

**Authors:** Joyce Noelly Vitor Santos, Elisângela Andrade Assis Madeira, Vanessa Gomes Brandão Rodrigues, Vitória Emanuelli Rodrigues Silva, Maria Cecília Sales Mendes Prates, Inara Caroline Marcelino Martins, Jéssica Stéfany Rocha, Vanessa Kelly da Silva Lage, Henrique Silveira Costa, Frederico Lopes Alves, Emílio Henrique Barroso Maciel, Ana Cristina Rodrigues Lacerda, Redha Taiar, Alessandro Sartorio, Pedro Henrique Scheidt Figueiredo, Vanessa Amaral Mendonça

**Affiliations:** 1 Universidade Federal dos Vales do Jequitinhonha e Mucuri, Programa de Pós-graduação em Reabilitação e Desempenho Funcional, Diamantina, MG, Brazil.; 2 Universidade Federal dos Vales do Jequitinhonha e Mucuri, Programa de Pós-graduação em Ciências da Saúde, Diamantina, MG, Brazil.; 3 Universidade Federal dos Vales do Jequitinhonha e Mucuri, Laboratório de Inflamação e Metabolismo, Diamantina, MG, Brazil.; 4 Université de Reims Champagne-Ardenne, Matériaux et Ingénierie Mécanique, Reims, France.; 5 Istituto Auxologico Italiano, Istituto di Ricovero e Cura a Carattere Scientifico, Experimental Laboratory for Auxo-Endocrinological Research, Piancavallo-Verbania, Italy.

**Keywords:** Functional Status, Activities of Daily Living, Kidney Diseases, Sustainable Development Goal 3 (SDG 3): Sustainable Development.

## Abstract

**Introduction:**

Hemodialysis patients present high morbidity and mortality rates. Functional capacity has emerged as a significant marker of mortality risk across populations, and the Glittre Activities of Daily Living (Glittre-ADL) test assesses it through tasks simulating daily activities. This study aimed to investigate the prognostic value of functional capacity, as assessed by the Glittre-ADL test, in hemodialysis patients.

**Methods:**

This was a prospective observational study conducted in a Brazilian reference hemodialysis center, involving patients aged 18 years or older who underwent dialysis three times weekly for at least three months. Baseline measurements were taken from April 2019 onward, and patients were followed until August 2024 to assess mortality. Clinical, anthropometric, and biochemical parameters were evaluated, and functional capacity was assessed through the Glittre-ADL test. Data were analyzed using Fine–Gray subdistribution hazards models, ROC curve analysis, Harrell’s C-index, and Kaplan–Meier survival analysis. Cutoff values were determined, and positive and negative predictive values were calculated. Confidence intervals were estimated using bootstrap resampling (1,000 replicates).

**Results:**

Seventy-six eligible patients were included. The median follow-up time was 38.33 (2.50–77.73) months. The Glittre-ADL test (HR: 1.003 [95%CI 1.001–1.006]; *p* = 0.03) and age (HR: 1.049 [95%CI 1.020–1.078]; *p* < 0.001) were independently associated with mortality. The optimal cutoff value for the Glittre-ADL test for identifying mortality risk was ≥268.5 seconds (AUC = 0.86; 95%CI 0.77–0.94; *p* < 0.0001).

**Conclusions:**

Performance in the Glittre-ADL test was associated with prognosis and accurately identified mortality risk among hemodialysis patients.

## INTRODUCTION

Hemodialysis is a type of renal replacement therapy that supplements the kidneys’ blood filtration function by utilizing artificial equipment to remove excess water, solutes, and toxins. This process maintains homeostasis in patients with end-stage renal disease (ESRD)^1^. The global prevalence of individuals requiring hemodialysis has increased significantly, primarily due to the growing prevalence of risk factors such as hypertension and diabetes^2,3^.

Although essential, both kidney failure and the treatment itself are associated with substantial symptom burden and high morbidity and mortality rates^2,3^. Hemodialysis patients frequently experience chronic fatigue, pain, depression^4,5^, and conditions such as bone disorders^6^, sarcopenia^7^, neuropathy^8^, and cardiovascular complications^9^. Moreover, these patients typically exhibit poor nutritional status due to the restrictive dietary regimen required and the reduced exercise capacity^10^, which leads to impaired physical functioning and severely affects motor skills and performance in essential activities of daily living (ADLs)^11^. These impairments contribute to functional decline, reduced quality of life, and worse clinical outcomes^11, 12, 13^.

ADLs encompass basic and instrumental tasks required for independent living and are influenced by cognitive, physical, and psychological factors^14^. Prior studies have shown that dependence in ADLs is associated with higher mortality among hemodialysis patients. However, these assessments have relied mainly on self-reported questionnaires^11,15^. While questionnaires indirectly assess the ability to perform ADLs through self-reporting, functional tests directly evaluate the physical functional capacity to perform these tasks^16^.

The Glittre Activities of Daily Living Test (Glittre-ADL test) has emerged as an assessment tool designed to evaluate the patients’ functional capacity by integrating lower- and upper-limb activities with a standardized set of tasks that simulate daily activities, such as walking, sitting, standing up, climbing stairs, reaching, gripping, and weight shifting^16^. The Glittre-ADL test is a validated tool closely associated with functional capacity and quality-of-life measures among hemodialysis patients^12,13^. However, its potential as a prognostic tool for mortality risk in this population has not yet been established. Importantly, predictive models for hemodialysis patients have traditionally been based solely on clinical and biochemical variables^17,18^, which may not fully capture other essential aspects of patients’ health. The incorporation of functional parameters into prognostic evaluation represents a novel and promising approach, with the potential to redefine risk stratification and improve clinical outcomes in this vulnerable population. This study aimed to investigate the prognostic value of functional capacity, as assessed by the Glittre-ADL test, for the early identification of mortality risk in hemodialysis patients.

## METHODS

### Study design

This prospective cohort study was conducted at a reference center for hemodialysis treatment and adhered to the ethical principles outlined in the Declaration of Helsinki. The study was approved by the Institutional Ethics Committee (CAAE: 60169822.1.0000.5108). Our research strategy followed the PROGRESS (Prognosis Research Strategy) recommendations^19^, and the STROBE Statement was used as a guide for reporting this study^20,21^.

### Participants

The inclusion criteria comprised patients aged 18 years or older who had been undergoing hemodialysis three times a week for at least three months. Exclusion criteria consisted of pregnancy, significant cognitive impairment, contraindications to functional testing, and inability to complete the assessments.

### Procedures

Baseline measurements were taken from April 2019 onward, and patients were followed until August 2024. Baseline assessments included anthropometric measurements, such as weight, height, and body mass index (BMI), as well as the Glittre-ADL test. These evaluations occurred before the second weekly hemodialysis session. After the therapy, body composition was measured using dual-energy X-ray absorptiometry (DEXA). Biochemical data were obtained from routine laboratory tests performed at the hemodialysis center and collected during the 15 days preceding the assessment week.

### Glittre-ADL test

The Glittre-ADL test was conducted as previously described and validated by Figueiredo et al.^13^ Participants began the test seated while carrying a backpack weighing 2.5 kg for women and 5 kg for men. From the sitting position, participants stood up and walked along a 10-m flat corridor, ascending and descending a two-step staircase (17 cm high and 27 cm deep) positioned midway along the path. They then continued walking the remaining 5 m to reach a shelf, where they were required to transfer three weights (each weighing 1 kg) from the upper shelf (at shoulder girdle level) to the lower shelf (at pelvic level) and then to the floor. Subsequently, they performed the reverse sequence, moving the weights back to the upper shelf and returning to the initial seated position to start the next lap immediately. Participants were instructed to complete five laps as quickly as possible without verbal encouragement. The total time required to complete the test was recorded.

### Known risk factors evaluated

Sex, age, hemodialysis vintage, presence of diabetes, anthropometric measurements, and biochemical variables (parathyroid hormone [PTH], sodium [Na], phosphorus [P], potassium [K], ferritin, and albumin) were evaluated due to their recognized prognostic value in hemodialysis patients^22^.

### Follow-up period and outcomes

The follow-up was initiated immediately after the baseline assessments and was conducted through regular visits by a researcher to the dialysis center. The endpoint was defined as death, regardless of the cause (clinical or surgical). Based on the outcome, participants were allocated to the non-survival or survival group. Causes of death were obtained from the electronic medical records of the dialysis unit.

### Sample size calculation

The OpenEpi software was used for the sample size calculation, with a significance level (alpha) of 5%, a statistical power of 95%, and an event rate of 30%. The *a priori* minimum sample size was set at 44 patients.

### Statistical analysis

Data analysis was performed using SPSS version 22.0 (SPSS Inc., Chicago, IL, USA) and MedCalc Statistical Software version 13.1 (MedCalc Software, Ostend, Belgium). The data distribution was assessed using the Kolmogorov–Smirnov test. Continuous variables were presented as mean ± standard deviation (for normally distributed data) or median and interquartile range (for non-normally distributed data). Group comparisons were performed through independent t-tests, the Mann–Whitney U test, or the chi-square test.

The association between the Glittre-ADL test and mortality was assessed using competing-risks analysis (Fine–Gray subdistribution hazards model), with transplantation as a competing event. The multivariable analysis was performed to assess the hazard ratio of the Glittre-ADL test for predicting mortality, adjusting for age, dialysis vintage, and diabetes^22^. As a sensitivity analysis, penalized Cox regression (ridge) was applied.

Receiver operating characteristic (ROC) analysis was performed to determine the sensitivity and specificity of different cutoff values of the Glittre-ADL test for predicting mortality. The area under the ROC curve (AUC) and its 95% confidence interval (CI) were calculated for all tests, and the optimal cutoff values were determined using the Youden index to identify the best combination of sensitivity and specificity. An AUC greater than 0.7 was considered acceptable, while an AUC greater than 0.8 was considered excellent for the proposed cutoff values^23^. Alternative cutoff points were proposed based on the trade-off between specificity and sensitivity. Positive predictive value (PPV) and negative predictive value (NPV) were calculated. Because mortality was analyzed as a time-to-event outcome, a complementary analysis using time-dependent ROC curves was also performed at predefined time horizons (12, 36, and 60 months). In addition, Harrell’s C-index was calculated from the Cox proportional hazards model to assess the model’s discriminatory power. The 95% confidence intervals for hazard ratios, AUCs, cutoff values, and Harrell’s C-index were estimated by bootstrap resampling (1,000 replicates). The Kaplan-Meier analysis was performed to assess cumulative survival across the Glittre-ADL categories.

## RESULTS

**Figure 1.**
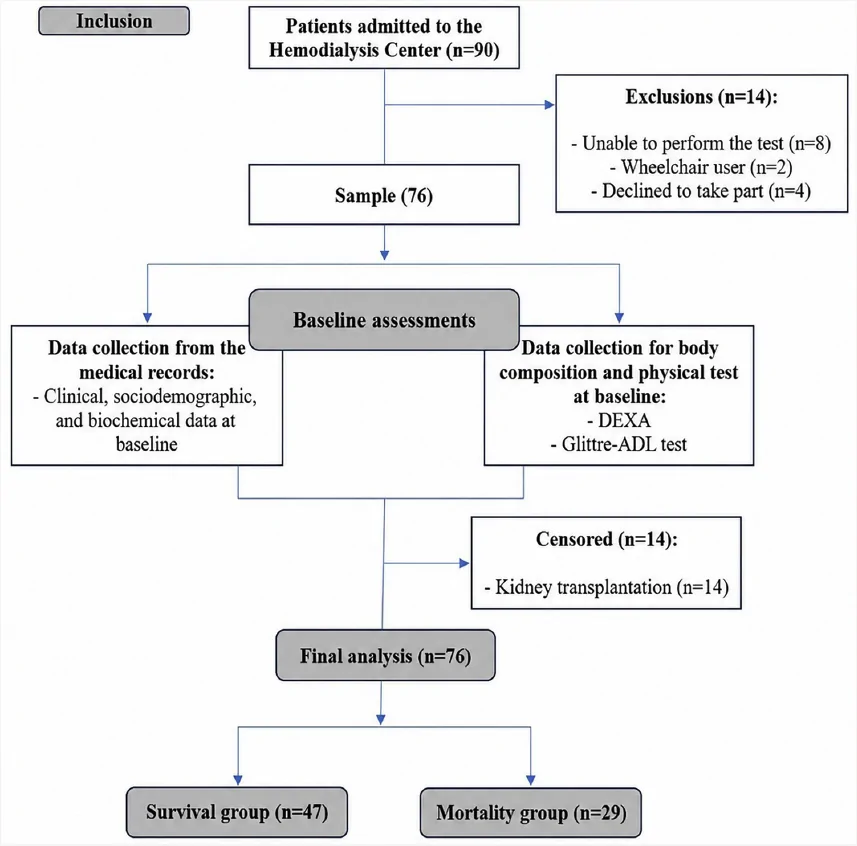
Flowchart of participant selection and follow-up. DEXA, dual-energy X-ray absorptiometry.

During the recruitment period, 90 patients were receiving hemodialysis. Seventy-six individualss were eligible for participation and assessed at baseline for clinical, sociodemographic, biochemical, and body composition variables, as well as for physical tests. Fourteen patients underwent kidney transplantation and were treated as competing events. Nevertheless, 76 patients were included in the study (Figure 1).

### Sample characteristics

At baseline, the mean age of the participants was 52.17 ± 15.91 years, and the sample was predominantly male (61%). The median hemodialysis vintage was 2.72 years (IQR 0.17–14.11), and the main known etiologies were hypertensive nephropathy (26%), diabetic nephropathy (19%), and chronic glomerulonephritis (9%). The median follow-up time was 38.33 months (IQR 2.50–77.73). Mortality occurred in 38% of the sample, and the causes of death included acute respiratory failure (21.5%), sudden death (18.4%), sepsis (13.8%), cancer (9.6%), stroke (4.9%), hemorrhagic shock (11.2%), and undetermined causes (20.6%). The sample was stratified into two groups based on the outcome: non-survival and survival. The characteristics of the groups are presented in Table 1.

**Table 1.** Comparison of demographic, biochemical, body composition, and Glittre-ADL test characteristics between the non-survival and survival groups.

Variables	All (n = 76)	Non-survival (n = 29)	Survival (n = 47)	*p*-value
Sex (M/F)	46 (61) / 30 (39)	16 (55) / 13 (45)	30 (64) / 17 (36)	0.77
Age (years)	52.17 ± 15.91	62.79 ± 12.61	45.62 ± 14.18	<0.001*
Dialysis vintage (years)	3.91 (3.22)	3.84 (4.01)	2.22 (5.24)	0.10
Diabetes, n (%)	17 (22)	9 (31)	8 (17)	0.28
**Biochemical**				
Vit D (ng/ml)	35.94 ± 15.26	32.21 ± 14.33	38.39 ± 15.51	0.86
PTH (pg/ml)	317.80 (285.00)	335.05 ± 223.66	350.84 ± 224.23	0.77
CRP (mg/L)	4.42 (7.00)	4.10 ± 9.26	4.10 (7.00)	0.49
Kt/V	1.46 ± 0.42	1.42 ± 0.23	1.49 ± 0.50	0.44
Hemoglobin (g/dL)	10.75 ± 1.49	10.60 ± 1.30	10.85 ± 1.59	0.47
Hematocrit (%)	33.34 ± 4.56	32.95 ± 3.60	34.75 (8.65)	0.57
Albumin (g/dL)	3.82 ± 0.37	3.70 ± 0.46	3.90 ± 0.30	0.06
Globulin (g/dL)	3.09 ± 0.40	3.15 ± 0.44	2.95 (0.40)	0.35
Potassium (mEq/L)	5.29 ± 0.67	5.26 ± 0.78	5.20 (0.80)	0.79
Sodium (mEq/L)	138.54 ± 2.65	137.90 ± 3.18	138.94 ± 2.21	0.13
Phosphorus (mg/dL)	4.66 ± 1.26	4.30 ± 0.98	4.45 (1.13)	0.06
Calcium (mg/dL)	9.05 ± 0.73	8.90 (0.60)	9.07 ± 0.77	0.76
LDL (mg/dL)	92.82 ± 29.38	94.56 ± 31.74	91.73 ± 28.17	0.71
HDL (mg/dL)	33.97 ± 9.04	34.59 ± 10.06	32.00 (12.25)	0.66
VLDL (mg/dL)	23.00 (18.75)	28.76 ± 15.99	21.00 (17.25)	0.14
Triglycerides (mg/dL)	116.00 (92.75)	178.41 ± 151.12	105.50 (86.75)	0.31
Ferritin (ng/mL)	368.00 (322.75)	1422.0 ± 413.31	395.00 (347.00)	0.97
Iron (μg/dL)	53.50 (33.75)	53.00 (34.50)	53.00 (34.25)	0.77
Alkaline phosphatase (U/L)	106.00 (80.50)	119.00 (111.50)	106.00 (84.50)	0.18
**Body composition**				
Dry weight (kg)	62.71 ± 13.44	61.34 ± 13.51	65.57 ± 13.47	0.48
WC (cm)	76.98 ± 25.62	76.38 ± 27.37	77.50 (25.00)	0.88
BMI (kg/m^2^)	24.20 (10.00)	22.80 (0.31)	32.19 ± 19.96	0.31
Body fat (%)	29.18 ± 10.43	29.79 ± 11.03	28.84 ± 10.19	0.71
ALM (kg)	18.09 ± 5.06	17.23 ± 4.94	19.56 ± 5.12	0.29
**Performance in ADL**				
Glittre-ADL (s)	240.50 (140.00)	364.0 ± 144.09	204.00 (71.00)	<0.001*

Abbreviations – Vit D: vitamin D; PTH: parathyroid hormone; CRP: C-reactive protein; Kt/V: fractional urea clearance; LDL: low-density lipoprotein; HDL: high-density lipoprotein; VLDL: very low-density lipoprotein; WC: waist circumference; BMI: body mass index; ALM: appendicular lean mass; ADL: activities of daily living.

*p < 0.05.

In the simple model, the Fine–Gray subdistribution hazards model analysis showed a significant association between the Glittre-ADL test and mortality (HR: 1.005 [95%CI 1.003–1.007]; *p* < 0.001). In the adjusted model, the Glittre-ADL test (HR: 1.003 [95%CI 1.001–1.008]; *p* = 0.031) and age (HR: 1.054 [95%CI 1.019–1.104]; *p* < 0.001) were independently associated with mortality risk (Table 2). The original Fine–Gray subdistribution hazards model analysis without bootstrapping is presented in the Supplementary Material (Table S1). Penalized Cox regression (ridge) is provided as a sensitivity analysis in the Supplementary Material (Table S2).

**Table 2.** Univariate and multivariate Fine–Gray subdistribution hazards model analysis of mortality.

Variables	Crude model			Adjusted model	
	HR (95%CI)	*p*-value		HR (95%CI)	*p*-value
Glittre-ADL test	1.005 (1.003–1.007)	<0.001	Glittre-ADL test	1.003 (1.001–1.008)	0.031
			Age (years)	1.054 (1.019–1.104)	<0.001
			Dialysis vintage (years)	1.013 (0.851–1.169)	0.778
			Diabetes (yes)	1.149 (0.390–1.299)	0.830

Abbreviations – HR: *hazard ratio*; CI: confidence interval.

Note – Adjusted for age, dialysis vintage, and diabetes. *Hazard ratio* (HR) and confidence interval calculated using bootstrapping (1,000 iterations). *p < 0,05.

**Figure 2.**
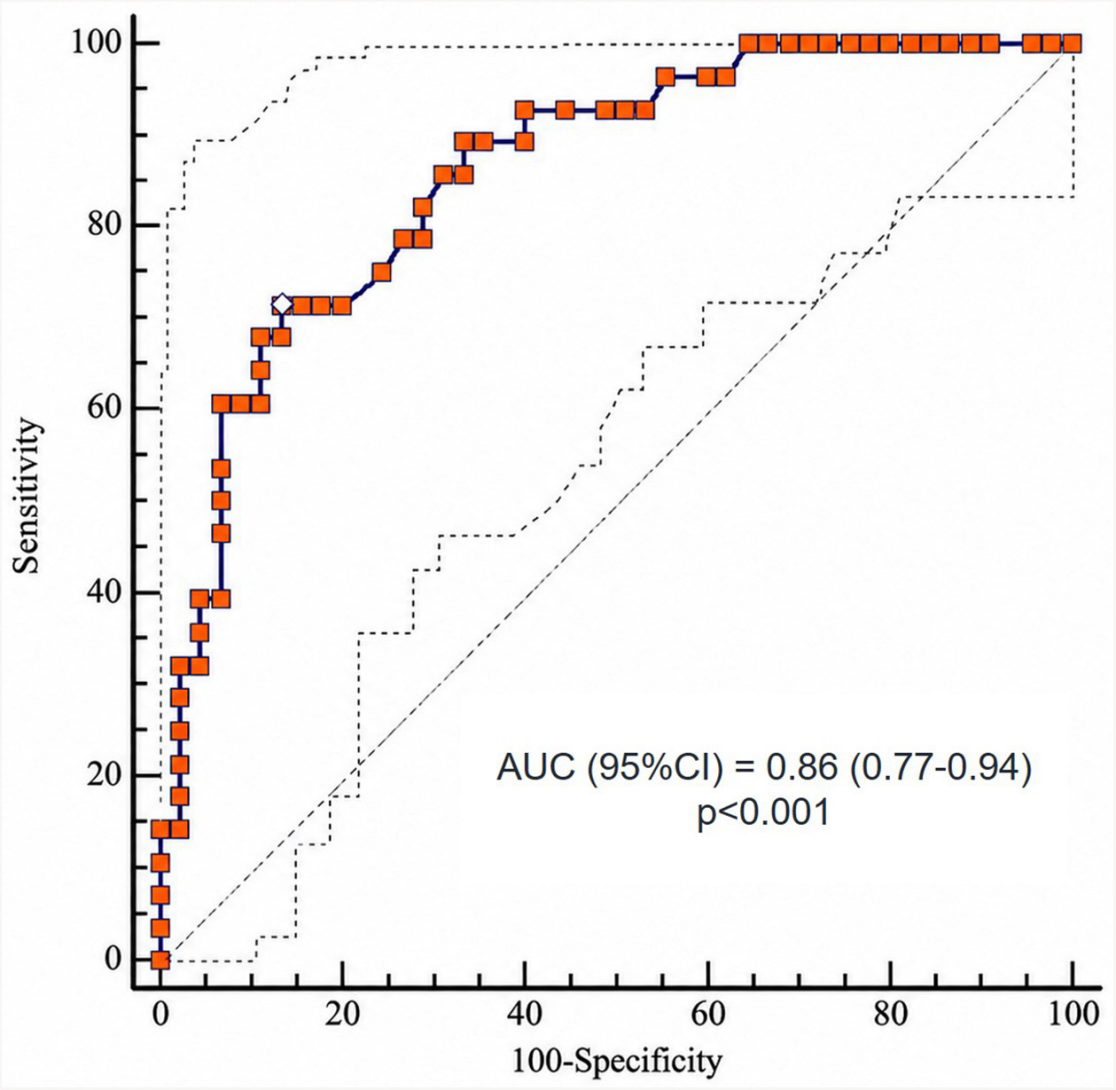
ROC curves for the Glittre-ADL test (AUC = 0.86 [0.77–0.94]; *p* < 0.0001) for mortality screening.

The area under the ROC curve (AUC) for identifying mortality risk using the Glittre-ADL was 0.86 (95%CI 0.77–0.94; *p* < 0.0001), indicating excellent accuracy (Figure 2). Table 3 presents the properties of the cutoff points, including sensitivity and specificity, as well as the test’s positive and negative predictive values for mortality risk screening.

**Table 3.** Cutoff points sensitivity, specificity, PPV, and NPV of the Glitre-ADL test for mortality risk screening.

Cutoff point	Sensitivity (95%CI)	Specificity (95%CI)	PPV (95%CI)	NPV (95%CI)
>193	100.0 (87.0–100.0)	35.6 (21.9–51.2)	49.1 (35.6–62.7)	100.0 (79.4–100.0)
>268.5*	71.4 (51.3–86.8)	86.7 (73.2–94.9)	76.9 (56.4–91.0)	83.0 (69.2–92.4)
>471	14.3 (4.0–32.7)	100.0 (92.1–100.0)	100.0 (39.8–100.0)	65.2 (52.8–76.3)

Abbreviations – PPV: positive predictive value; NPV: negative predictive value; CI: confidence interval.

Note – *Optimal cutoff value determined by the Youden index; bootstrapping (1,000 iterations) for confidence intervals.

Time-dependent ROC analysis showed AUC values of 0.70 (95%CI 0.51–0.90), 0.82 (95%CI 0.71–0.92), and 0.78 (95%CI 0.62–0.91) at 12-, 36-, and 60-month horizons, respectively (Figure S1). The Harrell’s C-index of the model was 0.77 (95%CI 0.70–0.85) (bootstrapping optimism-corrected C-index = 0.71; 95%CI 0.66–0.84), indicating moderate discriminatory ability.

**Figure 3.**
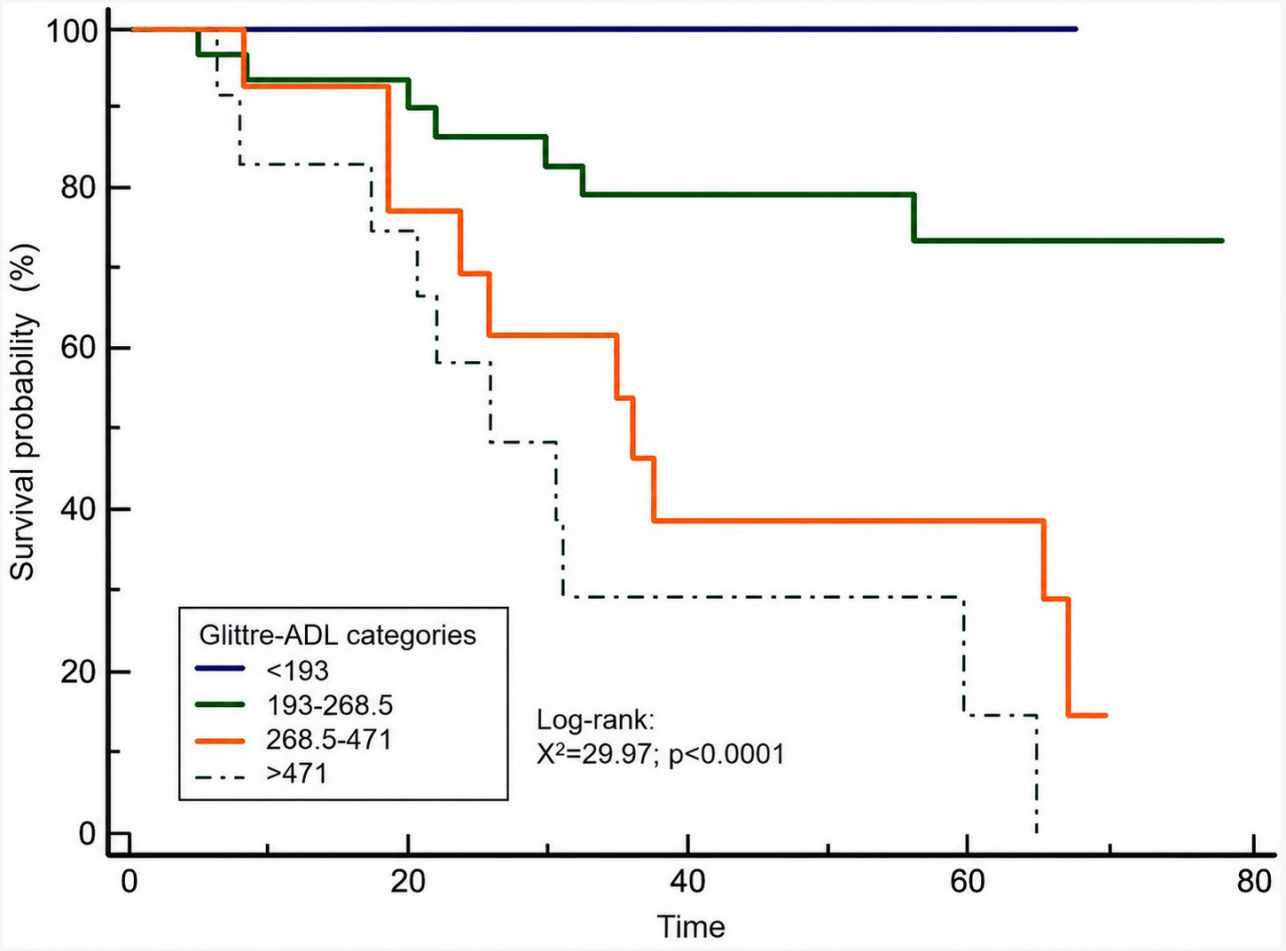
Kaplan-Meier estimate of cumulative survival by the Glittre-ADL test categories (<193 s, 193–268.5 s, 268.5–471 s; and >471 s).

Finally, the Kaplan-Meier curve showed that cumulative survival probabilities were significantly lower among hemodialysis patients with Glittre-ADL test performance times exceeding 193, 268.5, and 471 seconds, respectively (log-rank: χ^2^ = 29.97; *p* < 0.0001) (Figure 3).

## DISCUSSION

This study highlighted the association between functional capacity and mortality, as well as the prognostic value of the Glittre-ADL test in identifying mortality risk in hemodialysis patients. The main findings indicated that (1) hemodialysis patients who died during the follow-up period were older and had poorer performance on the Glittre-ADL test compared with those who survived; and (2) the Glittre-ADL test and age were independent predictors of mortality, even after adjusting for other factors, with excellent accuracy.

Both the Glittre-ADL test and age were independently associated with mortality. Each additional second on the test was associated with a 0.3% increase in risk (HR: 1.003 [95%CI 1.001–1.006]), indicating that poorer functional performance predicts a worse prognosis. This constitutes a modest but statistically significant increase in risk per unit of increase, and although the effect size per unit was small, the cumulative impact is clinically relevant. Age showed a stronger effect, with each additional year associated with a 4.9% increase in risk (HR: 1.049 [95%CI 1.020–1.078]).

Age is a well-established independent predictor of mortality in patients undergoing hemodialysis, with research indicating that physiological reserves decline, comorbidities increase, and the risk of mortality rises with advancing age^24, 25, 26, 27^. This association is multifactorial and reflects the cumulative burden of comorbidities, particularly cardiovascular disease, diabetes, and frailty, which are more prevalent with advancing age and substantially contribute to adverse outcomes^26,28^. In addition, aging is characterized by a progressive decline in physiological reserve, immunosenescence, and a chronic pro-inflammatory state, all of which increase susceptibility to infections and cardiovascular events^29, 30, 31^.

Notably, this study found that the Glittre-ADL test predicted mortality even in a relatively younger, non-elderly sample. This finding highlights the severity of this health condition, which leads to functional impairment and an increased risk of adverse outcomes even among younger individuals. It also underscores the importance of early risk identification, allowing for greater opportunity to modify the clinical course.

Still, Glittre-ADL was a predictor of mortality regardless of age, dialysis vintage, and the presence of diabetes. While some questionnaires subjectively assess independence in ADLs^32^, the Glittre-ADL test objectively evaluates functional status by measuring ADL performance through tasks that simulate typical everyday activities^12^. This test includes tasks such as sitting down and standing up from a chair, walking, carrying a bag, climbing stairs, moving objects with the upper limbs, and bending to pick up an object. Additionally, the test is repeated five times, allowing assessment of the patient’s functional and cardiorespiratory performance^12,13^. Still, the time to completion on the Glittre-ADL test was positively associated with moderate-to-vigorous daily physical activity measured by accelerometry, and the hemodynamic responses and perceived exertion were representative of what would be expected in submaximal exercise testing^12^. Thus, prior performance on this test may indicate a systemic deterioration and a reduction in the individuals’ functional reserve^13^, mainly associated with the musculoskeletal, neurological, metabolic, and cardiovascular systems. Furthermore, prior performance on the Glittre-ADL may indicate greater dependence and inactivity, which contribute to a poorer prognosis.

In the present study, the optimal cutoff value for Glittre-ADL in mortality screening was 268.5 seconds, with a sensitivity of 71.4%, a specificity of 86.7%, and an AUC of 0,86. Furthermore, the proposed cutoff point yielded a PPV of 76.9%, indicating that a test result of ≥ 268.5 seconds accurately predicts a mortality probability of only 76.9%. The NPV was 83.0%, indicating that a test result of < 268.5 seconds accurately predicts an 83.0% probability of survival. The high NPV reinforces the test’s utility as a reliable tool for ruling out mortality risk. In contrast, the more modest PPV suggests caution when using it to definitively identify high-risk patients.

Alternative cutoff points were proposed to explore different combinations of sensitivity and specificity and may be used as appropriate in clinical practice. The alternative cutoff point of 193 seconds had a sensitivity of 100% and an NPV of 100%. In clinical practice, this cutoff point is best suited for identifying individuals at low risk of mortality. Finally, the alternative cutoff of 471 seconds, with 100% specificity and 100% PPV, was more effective in identifying individuals at higher risk of mortality.

Although time-dependent ROC analysis is considered more appropriate for evaluating predictive performance in time-to-event data, the conventional ROC approach was retained to ensure comparability with the previous literature. Nevertheless, additional time-dependent analyses were conducted and are presented in the Supplementary Material to address this limitation.

This study has notable strengths that enhance its clinical and methodological significance. A key strength is the focus on functional capacity as a prognostic marker, an area that remains underexplored in the hemodialysis population. By emphasizing the predictive value of functional assessment, the study reinforces the importance of incorporating physical capacity measures into the routine evaluation and monitoring of these patients. This was the first study to explore the Glittre-ADL test, previously validated by Silva et al.^12^ and Figueiredo et al.^13^ in the hemodialysis population, and the first to evaluate its prognostic value.

### Limitations of the study

The cohort may not be highly representative of all hemodialysis centers. However, it is representative of dialysis centers with similar characteristics, such as those located in developing countries and in regions with a low Human Development Index (HDI) score.

### Implications

The Glittre-ADL test demonstrated substantial prognostic value in hemodialysis patients, reinforcing the importance of assessing functional capacity and supporting its use in preventive strategies and early mortality risk screening.

## CONCLUSION

Hemodialysis patients with poor performance on the Glittre-ADL test exhibited an increased risk of mortality, demonstrating that functional capacity is an important prognostic marker in this population. These findings underscore the importance of implementing strategies to evaluate and improve the functional capacity of hemodialysis patients, which may play a crucial role in improving their prognosis.

Moreover, functional capacity assessment represents a simple, rapid, and low-cost tool that should be increasingly considered in hemodialysis settings, including those in resource-limited regions.

## Conflict of interest

The authors declare no competing interests.

## Data Availability

The datasets generated and/or analyzed during the current study are available from the corresponding author upon reasonable request.
