# Supplementary material for: Prognostic value of functional capacity assessed by the Glittre Activities of Daily Living test in hemodialysis patients: a prospective observational study
Source: J Bras Nefrol. 2026 Sep 11;48(4):e20250316. doi: 10.1590/2175-8239-JBN-2025-0316en (PMC13565582; doi:10.1590/2175-8239-JBN-2025-0316en)
Supplement: Translation

# Translation: Valor prognóstico da capacidade funcional avaliada pelo teste Glittre de Atividades de Vida Diária (AVD-Glittre) em pacientes em hemodiálise: um estudo observacional prospectivo

**DOI:** 10.1590/2175-8239-JBN-2025-0316pt

**Authors:** Joyce Noelly Vitor Santos, Elisângela Andrade Assis Madeira, Vanessa Gomes Brandão Rodrigues, Vitória Emanuelli Rodrigues Silva, Maria Cecília Sales Mendes Prates, Inara Caroline Marcelino Martins, Jéssica Stéfany Rocha, Vanessa Kelly da Silva Lage, Henrique Silveira Costa, Frederico Lopes Alves, Emílio Henrique Barroso Maciel, Ana Cristina Rodrigues Lacerda, Redha Taiar, Alessandro Sartorio, Pedro Henrique Scheidt Figueiredo, Vanessa Amaral Mendonça

**Affiliations:** 1 Universidade Federal dos Vales do Jequitinhonha e Mucuri, Programa de Pós-graduação em Reabilitação e Desempenho Funcional, Diamantina, MG, Brasil.; 2 Universidade Federal dos Vales do Jequitinhonha e Mucuri, Programa de Pós-graduação em Ciências da Saúde, Diamantina, MG, Brasil.; 3 Universidade Federal dos Vales do Jequitinhonha e Mucuri, Laboratório de Inflamação e Metabolismo, Diamantina, MG, Brasil.; 4 Université de Reims Champagne-Ardenne, Matériaux et Ingénierie Mécanique, Reims, France.; 5 Istituto Auxologico Italiano, Istituto di Ricovero e Cura a Carattere Scientifico, Experimental Laboratory for Auxo-Endocrinological Research, Piancavallo-Verbania, Italy.

**Keywords:** Estado Funcional, Atividades Cotidianas, Nefropatias, Desenvolvimento Sustentável

## Abstract

**Introdução:**

Pacientes em hemodiálise apresentam elevadas taxas de morbidade e mortalidade. A capacidade funcional tem emergido como um importante marcador de risco de mortalidade em diferentes populações, e o teste Glittre de Atividades de Vida Diária (AVD-Glittre) a avalia por meio de tarefas que simulam atividades cotidianas. Este estudo teve como objetivo investigar o valor prognóstico da capacidade funcional, avaliada pelo teste de AVD-Glittre, em pacientes em hemodiálise.

**Métodos:**

Tratou-se de um estudo observacional prospectivo conduzido em um centro de referência em hemodiálise no Brasil, envolvendo pacientes com 18 anos ou mais, submetidos à diálise três vezes por semana por pelo menos três meses. As avaliações basais foram realizadas a partir de abril de 2019, e os pacientes foram acompanhados até agosto de 2024 para avaliação da mortalidade. Foram avaliados parâmetros clínicos, antropométricos e bioquímicos, e a capacidade funcional foi mensurada pelo teste de AVD-Glittre. Os dados foram analisados por meio de modelos de risco de subdistribuição de Fine–Gray, análise da curva ROC, índice C de Harrell e análise de sobrevida de Kaplan–Meier. Os valores de corte foram determinados, e os valores preditivos positivos e negativos foram calculados. Intervalos de confiança foram estimados por meio de reamostragem *bootstrap* (1.000 réplicas).

**Resultados:**

Setenta e seis pacientes elegíveis foram incluídos. O tempo mediano de seguimento foi de 38,33 (2,50–77,73) meses. O teste de AVD-Glittre (HR: 1,003 [IC95% 1,001–1,006]; *p* = 0,03) e a idade (HR: 1,049 [IC95% 1,020–1,078]; *p* < 0,001) estiveram independentemente associados à mortalidade. O ponto de corte ótimo do AVD-Glittre para identificação do risco de mortalidade foi ≥268,5 segundos (AUC = 0,86; IC95% 0,77–0,94; *p* < 0,0001).

**Conclusões:**

O desempenho no teste de AVD-Glittre associou-se ao prognóstico e identificou com acurácia o risco de mortalidade em pacientes em hemodiálise.

## INTRODUÇÃO

A hemodiálise é uma forma de terapia de substituição renal que complementa a função de filtração sanguínea dos rins, utilizando equipamentos artificiais para remover o excesso de água, solutos e toxinas. Esse processo mantém a homeostase em pacientes com doença renal em estágio terminal (DRET)^1^. A prevalência global de indivíduos que necessitam de hemodiálise tem aumentado significativamente, principalmente devido ao aumento da prevalência de fatores de risco, como hipertensão e diabetes^2,3^.

Tanto a insuficiência renal quanto o próprio tratamento estão associados, de forma cumulativa, a uma elevada carga de sintomas e a altas taxas de morbidade e mortalidade^2,3^. Pacientes em hemodiálise frequentemente apresentam fadiga crônica, dor, depressão^4,5^ e condições como distúrbios ósseos^6^, sarcopenia^7^, neuropatia^8^ e complicações cardiovasculares^9^. Além disso, esses pacientes geralmente apresentam estado nutricional comprometido devido ao regime alimentar restritivo exigido e à reduzida capacidade de se exercitar^10^, o que compromete a função física e afeta de forma significativa as habilidades motoras e o desempenho em atividades essenciais da vida diária (AVDs)^11^. Essas limitações contribuem para o declínio funcional, para a redução da qualidade de vida e para piores desfechos clínicos^11, 12, 13^.

As AVDs abrangem tarefas básicas e instrumentais necessárias à vida independente e são influenciadas por fatores cognitivos, físicos e psicológicos^14^. Estudos prévios demonstraram que a dependência nas AVDs está associada a maior mortalidade entre pacientes em hemodiálise. No entanto, essas avaliações têm se baseado principalmente em questionários autorreferidos^11,15^. Embora os questionários avaliem indiretamente a aptidão para AVDs por meio do autorrelato, os testes funcionais avaliam diretamente a capacidade física e funcional para a execução dessas tarefas^16^.

O teste de AVD-Glittre tem se consolidado como uma ferramenta de avaliação, desenvolvida para mensurar a capacidade funcional de pacientes por meio da integração das atividades dos membros inferiores e superiores em um conjunto padronizado de tarefas que simulam atividades cotidianas, como caminhar, sentar, levantar, subir escadas, alcançar, segurar e transferir peso^16^. O teste de AVD-Glittre é um instrumento validado, associado à capacidade funcional e a medidas de qualidade de vida em pacientes em hemodiálise^12,13^. No entanto, seu potencial como ferramenta prognóstica para predição do risco de mortalidade nessa população ainda não foi estabelecido. É importante destacar que os modelos preditivos para pacientes em hemodiálise têm sido tradicionalmente baseados exclusivamente em variáveis clínicas e bioquímicas^17,18^, o que pode não captar plenamente outros aspectos essenciais da saúde do paciente. A incorporação de parâmetros funcionais na avaliação prognóstica representa uma abordagem inovadora e promissora, com potencial para redefinir a estratificação de risco e melhorar os desfechos clínicos nessa população vulnerável. Assim, este estudo teve como objetivo investigar o valor prognóstico da capacidade funcional, avaliada pelo teste de AVD-Glittre, para a identificação precoce do risco de mortalidade em pacientes em hemodiálise.

## MÉTODOS

### Desenho do estudo

Tratou-se de um estudo observacional prospectivo conduzido em um centro de referência em hemodiálise, seguindo os princípios éticos estabelecidos na Declaração de Helsinki e aprovado pelo Comitê de Ética Institucional (CAAE: 60169822.1.0000.5108). Nossa estratégia de pesquisa seguiu as Recomendações PROGRESS (*Prognosis Research Strategy*)^19^, e a diretriz STROBE foi utilizada como guia para o relato deste estudo^20,21^.

### Participantes

Os critérios de inclusão compreenderam pacientes com idade igual ou superior a 18 anos, submetidos à hemodiálise três vezes por semana há pelo menos três meses. Os critérios de exclusão foram: gestação, déficit cognitivo significativo, contraindicações à realização de testes funcionais e incapacidade de completá-los.

### Procedimentos

As avaliações basais foram realizadas a partir de abril de 2019, e os pacientes foram acompanhados até dezembro de 2024. As avaliações iniciais incluíram medidas antropométricas, como peso, altura e índice de massa corporal (IMC), bem como o teste de AVD-Glittre. Essas avaliações foram realizadas antes da segunda sessão semanal de hemodiálise. Após a terapia, a composição corporal foi mensurada por meio da absorciometria por dupla emissão de raios X (DEXA). Os dados bioquímicos foram obtidos a partir de exames laboratoriais de rotina realizados no setor de hemodiálise, coletados durante os 15 dias que antecederam a semana de avaliação.

### Teste de AVD-glittre

O teste de AVD-Glittre foi conduzido conforme previamente descrito e validado por Figueiredo et al.^13^. Os participantes iniciaram o teste sentados, utilizando uma mochila com peso de 2,5 kg para as mulheres e de 5 kg para os homens. A partir da posição sentada, os indivíduos levantaram-se e caminharam ao longo de um corredor plano de 10 metros, subindo e descendo uma escada de dois degraus (17 cm de altura e 27 cm de largura), situada na metade do percurso. Em seguida, continuaram caminhando os 5 metros restantes até alcançar uma estante, onde deveriam transferir três pesos (cada um com 1 kg) da prateleira superior (na altura da cintura escapular) para a prateleira inferior (na altura pélvica) e, posteriormente, para o chão. Na sequência, realizaram o movimento inverso, retornando os pesos à prateleira superior e voltando à posição inicial, sentada, para iniciar imediatamente a próxima volta. Os participantes foram instruídos a completar cinco voltas o mais rápido possível, sem incentivo verbal. O tempo total de realização do teste foi registrado.

### Fatores de risco avaliados

Sexo, idade, tempo em hemodiálise, presença de diabetes, medidas antropométricas e variáveis bioquímicas (paratormônio [PTH], sódio [Na], fósforo [P], potássio [K], ferritina e albumina) foram avaliados devido ao seu reconhecido valor prognóstico em pacientes em hemodiálise^22^.

### Período de seguimento e desfechos

O seguimento foi iniciado imediatamente após as avaliações basais e conduzido por meio de visitas periódicas de um pesquisador ao centro de diálise. O desfecho foi definido como óbito, independentemente da causa (clínica ou cirúrgica). Com base no desfecho, os participantes foram alocados em dois grupos: óbito e sobreviventes. As causas de óbito foram obtidas a partir dos prontuários eletrônicos do setor.

### Cálculo do tamanho da amostra

O software *OpenEpi* foi utilizado para o cálculo do tamanho da amostra, com erro alfa de 5%, poder estatístico de 95% e taxa de eventos de 30%. O tamanho mínimo da amostra foi estimado *a priori* em 44 pacientes.

### Análise estatística

A análise dos dados foi realizada utilizando o SPSS, versão 22.0 (SPSS Inc., Chicago, IL, EUA), e o *MedCalc Statistical Software*, versão 13.1 (*MedCalc Software*, Ostend, Bélgica). A distribuição dos dados foi verificada por meio do teste de Kolmogorov–Smirnov. As variáveis contínuas foram apresentadas como média e desvio padrão (para distribuições normais) ou mediana e intervalo interquartil (para distribuições não normais). As comparações entre grupos foram realizadas por meio dos testes t independente, de Mann–Whitney ou do qui-quadrado.

A associação entre o teste de AVD-Glittre e a mortalidade foi avaliada por meio de uma análise de riscos competitivos (modelo de riscos de subdistribuição de Fine–Gray), considerando o transplante como evento competitivo. A análise multivariada foi realizada para avaliar o *hazard ratio* do teste de AVD-Glittre na predição de mortalidade, ajustando para idade, tempo em diálise e diabetes^22^. Como análise de sensibilidade, foi aplicada regressão de Cox penalizada (*ridge*).

A análise de *receiver operating characteristic* (ROC) foi realizada para determinar a sensibilidade e a especificidade de diferentes pontos de corte do teste AVD-Glittre na predição de mortalidade. A área sob a curva ROC (AUC) e seu intervalo de confiança de 95% (IC) foram calculados para todos os testes, e os pontos de corte ótimos foram determinados pelo índice de Youden, a fim de identificar a melhor combinação entre sensibilidade e especificidade. Uma AUC superior a 0,7 foi considerada aceitável, enquanto uma AUC superior a 0,8 foi considerada excelente para os pontos de corte sugeridos^23^. Pontos de corte alternativos foram propostos com base no equilíbrio entre especificidade e sensibilidade. Os valores preditivos positivos (VPP) e negativos (VPN) foram calculados. Como a mortalidade foi analisada como desfecho de tempo até o evento, também foi realizada uma análise complementar com curvas ROC dependentes do tempo, em horizontes temporais predefinidos (12, 36 e 60 meses). Além disso, o índice C de Harrell foi calculado com base no modelo de riscos proporcionais de Cox para avaliar a discriminação do modelo. Os intervalos de confiança de 95% dos *hazard ratios*, AUCs, pontos de corte e do índice C de Harrell foram estimados por meio de reamostragem *bootstrap* (1.000 réplicas). A análise de Kaplan–Meier foi realizada para avaliar a sobrevida cumulativa entre as categorias do AVD-Glittre.

## RESULTADOS

Durante o período de recrutamento, 90 pacientes estavam em hemodiálise. Setenta e seis indivíduos foram considerados elegíveis para participação e avaliados na linha de base quanto a variáveis clínicas, sociodemográficas, bioquímicas e de composição corporal, bem como quanto aos testes físicos. Quatorze pacientes receberam transplante renal e foram tratados, na análise estatística, como eventos competitivos. Ainda assim, todos os 76 pacientes foram incluídos no estudo (Figura 1).

**Figura 1.** Fluxograma de seleção e follow-up dos participantes. DEXA, absorciometria por dupla emissão de raios X.

### Características da amostra

Na linha de base, a idade média dos participantes foi de 52,17 ± 15,91 anos, e a amostra foi predominantemente do sexo masculino (61%). O tempo mediano em hemodiálise foi de 2,72 anos (IIQ = 0,17–14,11), e as principais etiologias conhecidas foram nefropatia hipertensiva (26%), nefropatia diabética (19%) e glomerulonefrite crônica (9%).

O tempo mediano de seguimento foi de 38,33 meses (IIQ = 2,50–77,73). A mortalidade ocorreu em 38% da amostra, e as causas de óbito incluíram insuficiência respiratória aguda (21,5%), morte súbita (18,4%), sepse (13,8%), câncer (9,6%), acidente vascular cerebral (4,9%), choque hemorrágico (11,2%) e causa indeterminada (20,6%).

A amostra foi estratificada em dois grupos, de acordo com o desfecho: óbito e sobreviventes. As características dos grupos estão apresentadas na Tabela 1.

**Tabela 1.** Comparação das características demográficas, bioquímicas, da composição corporal e do teste de AVD-Glittre entre os grupos óbito e sobreviventes.

Variáveis	Todos (n = 76)	Óbito (n=29)	Sobreviventes (n = 47)	Valor de *p*
Sexo (M/F)	46 (61) / 30 (39)	16 (55) / 13 (45)	30 (64) / 17 (36)	0,77
Idade (anos)	52,17 ± 15,91	62,79 ± 12,61	45,62 ± 14,18	<0,001*
Tempo de HD (anos)	3,91 (3,22)	3,84 (4,01)	2,22 (5,24)	0,10
Diabetes n (%)	17 (22)	9 (31)	8 (17)	0,28
**Bioquímicas**				
Vit D (ng/ml)	35,94 ± 15,26	32,21 ± 14,33	38,39 ± 15,51	0,86
PTH (pg/ml)	317,80 (285,00)	335,05 ± 223,66	350,84 ± 224,23	0,77
PCR (mg/L)	4,42 (7,00)	4,10 ± 9,26	4,10 (7,00)	0,49
Kt/V	1,46 ± 0,42	1,42 ± 0,23	1,49 ± 0,50	0,44
Hemoglobina (g/dL)	10,75 ± 1,49	10,60 ± 1,30	10,85 ± 1,59	0,47
Hematócrito (%)	33,34 ± 4,56	32,95 ± 3,60	34,75 (8,65)	0,57
Albumina (g/dL)	3,82 ± 0,37	3,70 ± 0,46	3,90 ± 0,30	0,06
Globulina (g/dL)	3,09 ± 0,40	3,15 ± 0,44	2,95 (0,40)	0,35
Potássio (mEq/L)	5,29 ± 0,67	5,26 ± 0,78	5,20 (0,80)	0,79
Sódio (mEq/L)	138,54 ± 2,65	137,90 ± 3,18	138,94 ± 2,21	0,13
Fósforo (mg/dL)	4,66 ± 1,26	4,30 ± 0,98	4,45 (1,13)	0,06
Cálcio (mg/dL)	9,05 ± 0,73	8,90 (0,60)	9,07 ± 0,77	0,76
LDL (mg/dL)	92,82 ± 29,38	94,56 ± 31,74	91,73 ± 28,17	0,71
HDL (mg/dL)	33,97 ± 9,04	34,59 ± 10,06	32,00 (12,25)	0,66
VLDL (mg/dL)	23,00 (18,75)	28,76 ± 15,99	21,00 (17,25)	0,14
Triglicérides (mg/dL)	116,00 (92,75)	178,41 ± 151,12	105,50 (86,75)	0,31
Ferritina (ng/mL)	368,00 (322,75)	1422,0 ± 413,31	395,00 (347,00)	0,97
Ferro (μg/dL)	53,50 (33,75)	53,00 (34,50)	53,00 (34,25)	0,77
Fosfatase alcalina (U/L)	106,00 (80,50)	119,00 (111,50)	106,00 (84,50)	0,18
**Composição corporal**				
Peso seco (kg)	62,71 ± 13,44	61,34 ± 13,51	65,57 ± 13,47	0,48
CA (cm)	76,98 ± 25,62	76,38 ± 27,37	77,50 (25,00)	0,88
IMC (kg/m^2^)	24,20 (10,00)	22,80 (0,31)	32,19 ± 19,96	0,31
Gordura corporal (%)	29,18 ± 10,43	29,79 ± 11,03	28,84 ± 10,19	0,71
MMA (kg)	18,09 ± 5,06	17,23 ± 4,94	19,56 ± 5,12	0,29
**Desempenho nas AVDs**				
AVD-Glittre (s)	240,50 (140,00)	364,0 ± 144,09	204,00 (71,00)	<0,001*

Abreviaturas – HD: hemodiálise; Vit D: vitamina D; PTH: paratormônio; PCR: proteína C reativa; Kt/V: depuração fracionada de ureia; LDL: lipoproteína de baixa densidade; HDL: lipoproteína de alta densidade; VLDL: lipoproteína de muito baixa densidade; CA: circunferência abdominal; IMC: índice de massa corporal; MMA: massa magra apendicular; AVDs: atividades de vida diária.

*p < 0,05.

No modelo simples, a análise por meio do modelo de riscos de subdistribuição de Fine–Gray demonstrou associação significativa entre o teste de AVD-Glittre e a mortalidade (HR: 1,005 [IC95% 1,003–1,007]; *p*<0,001).

No modelo ajustado, o AVD-Glittre (HR: 1,003 [IC95% 1,001–1,008]; *p*=0,031) e a idade (HR: 1,054 [IC95% 1,019–1,104]; *p*<0,001) estiveram independentemente associados ao risco de mortalidade (Tabela 2).

**Tabela 2.** Análise univariada e multivariada do modelo de riscos de subdistribuição de Fine–Gray para mortalidade.

Variáveis	Modelo simples			Modelo ajustado	
	HR (IC95%)	Valor de *p*		HR (IC95%)	Valor de *p*
Teste de AVD-Glittre	1,005 (1,003–1,007)	<0,001	Teste de AVD-Glittre	1,003 (1,001–1,008)	0,031
			Idade (anos)	1,054 (1,019–1,104)	<0,001
			Tempo de hemodiálise (anos)	1,013 (0,851–1,169)	0,778
			Diabetes (sim)	1,149 (0,390–1,299)	0,830

Abreviaturas – HR: *hazard ratio*; IC: intervalo de confiança.

Nota – Ajustado para idade, tempo de hemodiálise e diabetes. *Hazard ratio* (HR) e intervalo de confiança foram calculados por meio de reamostragem *bootstrap* (1.000 iterações). *p < 0,05.

A análise original do modelo de riscos de subdistribuição de Fine–Gray, sem reamostragem *bootstrap*, está descrita no Material Suplementar (Tabela S1). A regressão de Cox penalizada (*ridge*) é apresentada como análise de sensibilidade no Material Suplementar (Tabela S2).

A área sob a curva ROC (AUC) para identificação do risco de mortalidade por meio do AVD-Glittre foi de 0,86 (IC95% 0,77–0,94; *p*<0,0001), indicando boa acurácia (Figura 2). A Tabela 3 apresenta as propriedades dos pontos de corte, incluindo sensibilidade e especificidade, bem como os valores preditivos positivos e negativos do teste na triagem de mortalidade.

**Figura 2.** Curvas ROC do teste de AVD-Glittre (AUC = 0,86 [IC95% 0,77–0,94]; *p* < 0,0001) para triagem de mortalidade.

**Tabela 3.** Pontos de corte sensibilidade, especificidade, valor preditivo positivo (VPP) e valor preditivo negativo (VPN) do teste de AVD-Glittre na triagem do risco de mortalidade.

Pontos de corte	Sensibilidade (IC95%)	Especificidade (IC95%)	VPP (IC95%)	VPN (IC95%)
>193	100,0 (87,0–100,0)	35,6 (21,9–51,2)	49,1 (35,6–62,7)	100,0 (79,4–100,0)
>268,5*	71,4 (51,3–86,8)	86,7 (73,2–94,9)	76,9 (56,4–91,0)	83,0 (69,2–92,4)
>471	14,3 (4,0–32,7)	100,0 (92,1–100,0)	100,0 (39,8–100,0)	65,2 (52,8–76,3)

Abreviaturas – VPP: valor preditivo positivo; VPN: valor preditivo negativo; IC: intervalo de confiança. *

Nota – Ponto de corte ótimo determinado pelo índice de *Youden*; reamostragem *bootstrap* (1.000 iterações) para os intervalos de confiança.

A análise de curvas ROC dependentes do tempo demonstrou valores de AUC de 0,70 (IC95% 0,51–0,90), 0,82 (IC95% 0,71–0,92) e 0,78 (IC95% 0,62–0,91) nos horizontes de 12, 36 e 60 meses, respectivamente (Figura S1). O índice C de Harrell do modelo foi de 0,77 (IC95% 0,70–0,85) (índice C corrigido por reamostragem *bootstrap* = 0,71; IC95% 0,66–0,84), indicando capacidade discriminativa moderada.

Por fim, na análise de Kaplan–Meier, as probabilidades cumulativas de sobrevida foram significativamente menores entre os pacientes em hemodiálise com desempenho no teste de AVD-Glittre superior a 193 s, 268,5 s e 471 s, respectivamente (teste de *log-rank*: χ^2^ = 29,97; *p* < 0,0001) (Figura 3).

**Figura 3.** Estimativa de Kaplan–Meier da sobrevida cumulativa por categorias do teste de AVD-Glittre (<193; 193–268,5; 268,5–471; e >471 s).

## DISCUSSÃO

Este estudo evidenciou a associação entre a capacidade funcional e a mortalidade, bem como o valor prognóstico do teste de AVD-Glittre na identificação do risco de mortalidade em pacientes em hemodiálise. Os principais achados indicaram que: (1) pacientes em hemodiálise que evoluíram para óbito durante o período de seguimento apresentaram maior idade e pior desempenho no teste de AVD-Glittre em comparação com aqueles que sobreviveram; e (2) o teste de AVD-Glittre e a idade foram preditores independentes de mortalidade, mesmo após o ajuste para outros fatores, com boa acurácia.

Tanto o teste de AVD-Glittre quanto a idade apresentaram uma associação independente com a mortalidade. Cada segundo adicional no teste esteve associado a um aumento de 0,3% no risco (HR: 1,003 [IC95% 1,001–1,006]), indicando que pior desempenho funcional prediz pior prognóstico. Trata-se de um aumento modesto, porém estatisticamente significativo, no risco por unidade de incremento e, embora o tamanho do efeito por unidade seja pequeno, o impacto cumulativo é clinicamente relevante. A idade apresentou um efeito mais pronunciado, com cada ano adicional associado a um aumento de 4,9% no risco (HR: 1,049 [IC95% 1,020–1,078]).

A idade é um preditor independente bem estabelecido da mortalidade em pacientes em hemodiálise, com evidências de que as reservas fisiológicas diminuem, as comorbidades aumentam e o risco de mortalidade aumenta à medida que a idade avança^24, 25, 26, 27^. Essa associação é multifatorial e reflete a carga cumulativa de comorbidades, particularmente doenças cardiovasculares, diabetes e fragilidade, que se tornam mais prevalentes com o envelhecimento e contribuem substancialmente para desfechos adversos^26,28^. Além disso, o envelhecimento é caracterizado por declínio progressivo da reserva fisiológica, imunossenescência e um estado inflamatório crônico de baixo grau, fatores que aumentam a suscetibilidade a infecções e eventos cardiovasculares^29, 30, 31^.

Notavelmente, este estudo demonstrou que o teste de AVD-Glittre foi capaz de predizer a mortalidade mesmo em uma amostra relativamente mais jovem, composta por indivíduos não idosos. Esse achado evidencia a gravidade dessa condição de saúde, que leva ao comprometimento funcional e ao aumento do risco de desfechos adversos mesmo entre indivíduos mais jovens. Além disso, reforça a importância da identificação precoce do risco, o que permite maior oportunidade de modificar o curso clínico. Ainda assim, o AVD-Glittre manteve-se como preditor de mortalidade, independentemente do tempo em hemodiálise e da presença de diabetes. Enquanto alguns questionários avaliam, de forma subjetiva, a independência nas AVDs^32^, o teste de AVD-Glittre avalia objetivamente o estado funcional ao mensurar o desempenho nessas atividades por meio de tarefas que simulam atividades cotidianas^12^. Esse teste inclui tarefas como sentar e levantar de uma cadeira, caminhar, carregar uma bolsa, subir escadas, mover objetos com os membros superiores e inclinar-se para pegar um objeto.

Adicionalmente, o teste é repetido cinco vezes, permitindo a avaliação do desempenho funcional e cardiorrespiratório do paciente^12,13^. Ainda, o tempo de execução no AVD-Glittre apresentou associação positiva com a atividade física diária de intensidade moderada a vigorosa, mensurada por acelerometria, e as respostas hemodinâmicas e a percepção de esforço foram representativas do esperado em testes de exercício submáximo^12^. Assim, o desempenho prévio nesse teste pode indicar uma deterioração sistêmica e uma redução da reserva funcional dos indivíduos^13^, principalmente associadas aos sistemas muscular, ósseo, neurológico, metabólico e cardiovascular. Além disso, o desempenho no AVD-Glittre pode refletir maior dependência funcional e inatividade, fatores que contribuem para um pior prognóstico.

No presente estudo, o ponto de corte ótimo do AVD-Glittre para triagem de mortalidade foi de 268,5 segundos, com sensibilidade de 71,4%, especificidade de 86,7% e AUC de 0,86. Além disso, o ponto de corte proposto apresentou valor preditivo positivo (VPP) de 76,9%, indicando que um resultado ≥ 268,5 segundos está associado a uma probabilidade de mortalidade de 76,9%. O valor preditivo negativo (VPN) foi de 83,0%, indicando que um resultado < 268,5 segundos está associado a uma probabilidade de sobrevida de 83,0%. O elevado VPN reforça a utilidade do teste como uma ferramenta confiável para descartar o risco de mortalidade. Em contrapartida, o VPP mais discreto sugere cautela ao utilizá-lo para identificar, de forma definitiva, pacientes de alto risco.

Pontos de corte alternativos foram propostos para explorar diferentes combinações entre sensibilidade e especificidade, podendo ser utilizados conforme o contexto clínico. O ponto de corte alternativo de 193 segundos apresentou sensibilidade de 100% e VPN de 100%. Na prática clínica, esse ponto de corte é mais adequado para identificar indivíduos com baixo risco de mortalidade. Por fim, o ponto de corte alternativo de 471 segundos, com especificidade de 100% e VPP de 100%, mostrou-se mais eficaz na identificação de indivíduos com maior risco de mortalidade.

Embora a curva ROC dependente do tempo seja mais apropriada para avaliar o desempenho preditivo em dados de tempo até o evento, a abordagem convencional de ROC foi mantida para garantir comparabilidade com a literatura prévia. Ainda assim, análises adicionais, dependentes do tempo, foram realizadas e apresentadas no Material Suplementar, a fim de mitigar essa limitação.

Este estudo apresenta pontos fortes importantes que reforçam sua relevância clínica e metodológica. Um dos principais pontos fortes é o enfoque na capacidade funcional como marcador prognóstico, um campo ainda pouco explorado na população em hemodiálise. Ao enfatizar o valor prognóstico da avaliação funcional, o estudo reforça a importância de incorporar medidas de capacidade física na avaliação e no monitoramento rotineiro desses pacientes. Além disso, este foi o primeiro estudo a explorar o teste de AVD-Glittre, previamente validado por Silva et al.^12^ e Figueiredo et al.^13^ na população em hemodiálise, e o primeiro a avaliar seu valor prognóstico.

### Limitações do estudo

A amostra deste estudo pode não ser altamente representativa de todos os centros de hemodiálise. No entanto, é representativa de centros de diálise com características semelhantes, como os localizados em países em desenvolvimento e em regiões com baixo Índice de Desenvolvimento Humano (IDH).

### Implicações

O teste de AVD-Glittre demonstrou valor prognóstico relevante em pacientes em hemodiálise, reforçando a importância da avaliação da capacidade funcional nessa população e sustentando sua utilização em estratégias preventivas e na triagem precoce do risco de mortalidade.

## CONCLUSÃO

Pacientes em hemodiálise com pior desempenho no teste de AVD-Glittre apresentaram maior risco de mortalidade, evidenciando que a capacidade funcional é um importante marcador prognóstico nessa população. Esses achados reforçam a importância da implementação de estratégias para avaliar e melhorar a capacidade funcional dos pacientes em hemodiálise, o que pode desempenhar papel crucial na melhora do prognóstico clínico. Além disso, a avaliação funcional é uma ferramenta simples, rápida e de baixo custo, que deve ser implementada na prática clínica em hemodiálise, inclusive em regiões com recursos limitados.

## Conflito de interesse

Os autores declaram não haver conflitos de interesse.

## Data Availability

Os conjuntos de dados gerados e/ou analisados no presente estudo estão disponíveis junto ao autor correspondente, mediante solicitação razoável.
